# The CTLH Complex in Cancer Cell Plasticity

**DOI:** 10.1155/2019/4216750

**Published:** 2019-11-30

**Authors:** Nickelas Huffman, Dario Palmieri, Vincenzo Coppola

**Affiliations:** Department of Cancer Biology and Genetics, The Ohio State University Comprehensive Cancer Center, Columbus, Ohio 43210, USA

## Abstract

Cancer cell plasticity is the ability of cancer cells to intermittently morph into different fittest phenotypic states. Due to the intrinsic capacity to change their composition and interactions, protein macromolecular complexes are the ideal instruments for transient transformation. This review focuses on a poorly studied mammalian macromolecular complex called the CTLH (carboxy-terminal to LisH) complex. Currently, this macrostructure includes 11 known members (ARMC8, GID4, GID8, MAEA, MKLN1, RMND5A, RMND5B, RANBP9, RANBP10, WDR26, and YPEL5) and it has been shown to have E3-ligase enzymatic activity. CTLH proteins have been linked to all fundamental biological processes including proliferation, survival, programmed cell death, cell adhesion, and migration. At molecular level, the complex seems to interact and intertwine with key signaling pathways such as the PI3-kinase, WNT, TGF*β*, and NF*κ*B, which are key to cancer cell plasticity. As a whole, the CTLH complex is overexpressed in the most prevalent types of cancer and may hold the key to unlock many of the biological secrets that allow cancer cells to thrive in harsh conditions and resist antineoplastic therapy.

## 1. Introduction

Cancer cell heterogeneity and plasticity are the two major obstacles to the cure of cancer [1, 2]. They are intricately linked but plasticity, which takes advantage of a variety of physiological programs, is considered the main reason why cancer is a quickly moving target [3–5].

Broadly speaking, plasticity can be defined as the ability of tumor cells to adapt to adverse conditions and evolve, implementing advantageous phenotypic changes [3, 6]. This adaptability is key to survival and proliferation in harsh conditions and involves fast and reversible rewiring of cellular networks and signaling pathways. Indeed, cancer cells can aberrantly turn on and off pathways that are otherwise transiently needed during organism development [7].

The typical example of cancer cell plasticity is the epithelial to mesenchymal transition (EMT), which is necessary for cell migration and secondary localization of solid epithelial malignancies. However, in order to establish the new colony when tumor cells reach their destination, a reverse mesenchymal to epithelial transition (MET) ensues [8–10]. In some instances, cancer cells may retain phenotypic characteristics that are common to both the epithelial and the mesenchymal states being more tumorigenic and drug resistant [9, 11–13]. Likewise, something similar to the EMT-MET changes is likely to happen when cells are subject to specific types of stress like treatments with anticancer drugs. Obviously, reciprocal communication between cancer cells and the microenvironment is crucial in determining the fittest phenotype [14–19]. Also, the existence of micro-environmental niches enabling survival and proliferation of malignant cells has emerged together with the concept that, within a tumor mass, some cells combine self-renewal with increased plasticity [20]. Tumor cells with increased self-renewal capacity abundant in advanced stages of disease are major contributors to tumor heterogeneity and resistance to therapy [21]. Most importantly, these cells are endowed with abnormally activated pathways such as PI3-kinase, WNT, and TGF*β*, all of which are intimately linked to self-renewal and plasticity itself [22, 23]. In fact, targeting these pathways has become a major goal for therapy [24, 25].

The cellular changes at the basis of plasticity are not “fixed” and can be of epigenetic nature. Without mutating the genome, cells can use these mechanisms to turn on and off specific genes [26]. However, epigenetic changes also have a certain degree of irreversibility and might not always be enacted fast enough to respond to acute harmful threats. Therefore, cells can use a type of change that is faster and based on rewiring mediated by quick protein posttranslational modifications. Regardless of the mechanism, cells will need to ultimately coordinate the changes in global signaling and concomitantly resolve potential incompatibilities.

Macromolecular complexes connected to a variety of signaling pathways are best positioned to act as fast coordinators of cell plasticity. In fact, they can quickly reshape and alter their member composition, implementing changes in a very efficient and rapid manner. The study of their functions is likely to be the next frontier in cancer biology.

Specifically, this review focuses on a poorly studied multisubunit structure called the CTLH (carboxy-terminal to LisH) complex and its links with a variety of signaling pathways and fundamental biological processes at the basis of cancer cell plasticity.

The composition of this structure may be variable and dynamically different depending on cell conditions and/or tissue-specific features. Ultimately, due also to its localization in both nucleus and cytoplasm, the CTLH complex is perfectly poised to integrate the myriad of different extracellular cues and coordinate the appropriate quick and reversible cellular changes necessary for cancer cells to thrive and become resistant to therapy.

## 2. CTLH Complex as Mammalian Ortholog of the Yeast GID Complex

**Figure 1:**
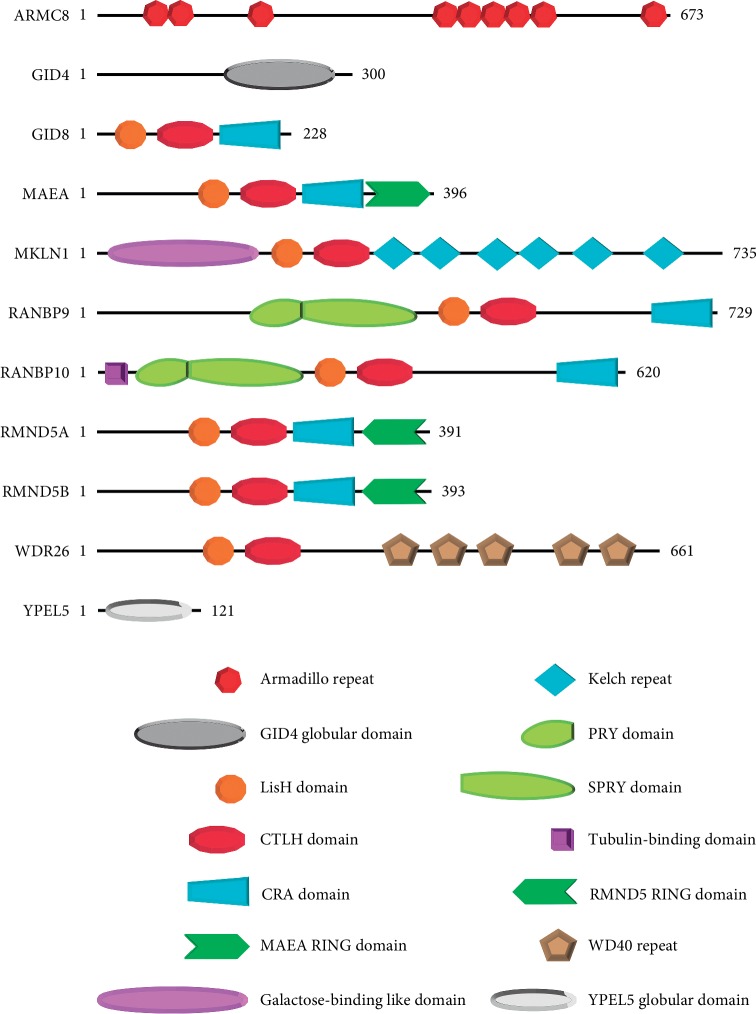
Schematic representation of the 11 members of the mammalian CTLH complex. Except for ARMC8, GID4, and YPEL5, all the other members present a LisH domain followed by a defining CTLH domain. Both LisH and CTLH domains are considered protein-protein interaction domains. MAEA in association with RMND5A or RMND5B provides the E3-ligase enzymatic activity of the complex. RANBP9 and RANBP10 are collectively called Scorpins (Spry-COntaining Ran binding ProteINS).

The mammalian CTLH complex derives its name from a protein-protein interaction domain shared by the majority of its core members (Figure 1). This multimolecular structure is the evolutionary equivalent of the yeast GID complex (Table 1) [27, 28]. As a whole functional unit, this complex has been studied mainly in *S. cerevisiae* and shown to respond to nutrient changes in the microenvironment [29]. Interestingly, whilst following a short-term starvation from glucose, the entire complex implements degradation of gluconeogenesis enzymes by proteasome-mediated mechanisms, some of its members are enacting an autophagy-mediated degradation of the same enzymes when the starvation is prolonged [29, 30]. Therefore, the equivalent of the CTLH complex confers to yeast the ability to adapt to both mild and severe changes of nutrient availability using a different configuration.

### 2.1. Definition of the CTLH Complex

**Figure 2:**
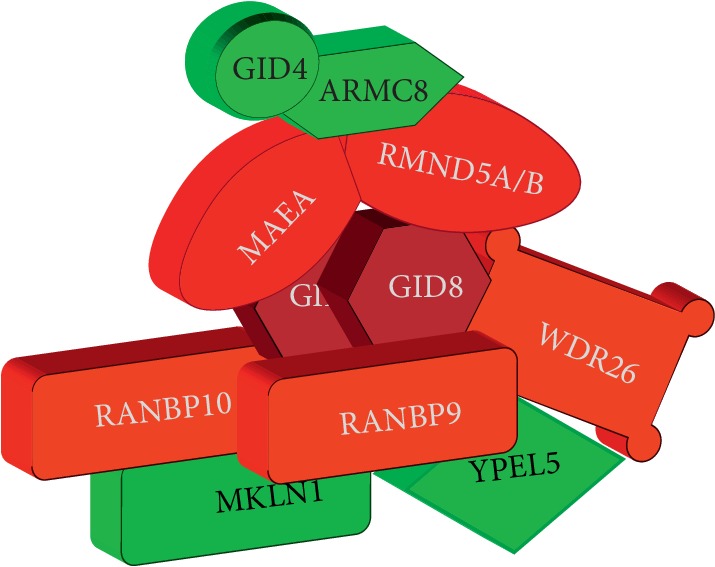
Schematic representation of the CTLH complex. The mammalian CTLH complex is a heterodecameric multimolecular structure built on dimer of GID8 (DARK RED). Its core (RED) includes the heterodimer MAEA-RMND5A or MAEA-RMND5B that together provide the E3-ligase enzymatic activity, the Scorpins (RANBP9, RANBP10), and WDR26. Peripheral components (GREEN) are GID4, ARMC8, MKLN1, and YPEL5.

It has been recently established that the CTLH complex is a heterodecameric molecular aggregate built on a GID8 dimer (Figure 2) [31]. According to data, there is a core of six CTLH proteins plus additional four peripheral members. Although this is not definitively proven, the known topology of the yeast complex, the ability of GID8 to self-dimerize, and the fact that GID8 is also the smallest of the proteins found in the CTLH core complex all are in agreement with this model [32, 33].

Based on the analysis of the yeast GID complex, RANBP9 too is considered essential for the assembly of the CTLH macrostructure [29]. Therefore, both GID8 and RANBP9 are necessary for a fully functional CTLH complex. Notably, the yeast equivalent of RANBP9 called Gid1 has evolved in humans into two different genes with high homology: RANBP9 and RANBP10 (a.k.a. Scorpins) [34–37]. Both of them seem to be present at the same time in the CTLH complex and it is conceivable that they have, at least in part, overlapping functions [35]. However, RANBP10 has been shown to inhibit the pro-proliferative effects mediated by RANBP9 on the tyrosine kinase signaling pathway [38]. Also, the two respective KO mice have different phenotypes [39–42].

In addition to GID8, RANBP9, and RANBP10, proteomic and biochemistry evidence shows that the heterodimer MAEA-RMND5 is an integral part of the core of the CTLH structure. RMND5 has evolved from the yeast Gid2 into two paralogs bearing an atypical RING domain, RMND5A and RMND5B, and they are the necessary partners for MAEA to confer E3-ligase activity to the CTLH complex [43]. The recent study from Lampert et al. indicated that they are mutually exclusive in the formation of the CTLH complex [31]. This mutual exclusivity suggests the existence of at least two different CTLH complexes (one with RMND5A and one with RMND5B) that might be in equilibrium within the same cells likely conferring different substrate specificities and providing an additional layer of fine-tuning of the E3-ligase activity [44].

Finally, part of the core is also considered WDR26, which binds to RANBP9 [28, 31, 45, 46]. On the other hand, GID4 together with ARMC8, MKLN1, and YPEL5 are the peripheral component [31]. To date, it is still not clear whether MKLN1 is a protein integral part of the complex since it does not have an accepted equivalent in the GID complex. Recently, it has been proposed that MKLN1 is part of the complex but also a substrate of the E3-ligase MAEA-RMND5 heterodimer [28].

A recent paper questioned whether ARMC8 too is the human ortholog of yeast Gid5 [47]. However, it is not disputed that ARMC8 is part of the human CTLH complex [45, 46]. Finally, YPEL5 presents sequence similarities with the yeast protein Moh1p, which is considered a nonessential component of the GID complex [48].

### 2.2. Known Functions of the Mammalian CTLH Complex

As single entities, CTLH members have been shown to be involved in a variety of processes [27, 49, 50]. Some of them have been linked to intellectual disability, neurodegenerative disease, and personality disorders [50–55]. CTLH proteins have also been involved in development and function of specific cell populations and tissues [39, 40, 42, 56–58]. These physiological or noncancer-related roles are outside the scope of this review.

One common theme in cancer-relevant systems is the ability of CTLH proteins to influence the abundance, stability, and subcellular localization of other proteins [27, 49]. However, it is not known if this is accomplished by always modulating the degradation mediated by the CTLH complex itself or through yet unknown mechanisms. Similarly, it is not clarified yet whether the E3-ligase activity of the complex is always involved in the phenotypes observed in cancer.

The topology of the complex appears to be evolutionarily conserved and the interactions between different members are similar in humans compared to yeast (Figure 2 and Table 1) [28, 31]. Observed differences in the composition are mainly due to the evolutionary duplication of Gid1 into the two paralogs RANBP9 and RANBP10 and Gid2 also into two paralogs, RMND5A and RMND5B [31].

To date, one main difference between the mammalian CTLH and the yeast GID might be functional. The latter has been shown to mediate the degradation of enzymes no longer required for glucose synthesis while there is only limited evidence showing that in kidney cells, the mammalian CTLH performs a similar function by binding to Bicaudal C1 [59]. A recent article showed that overexpression of MAEA in mouse hepatocytes lowers gluconeogenesis [60]. However, the CTLH complex may not be able to always regulate gluconeogenesis in all cell types [31]. Although this needs to be confirmed, it suggests that the CTLH complex may have functionally diverged and acquired different functions compared to the yeast GID. Understandably, nutrient availability is only one component of the extracellular cues that cells have to respond to in multicellular organisms. In fact, members of the CTLH complex have been linked to response to a variety of growth factors and hormones that mediate intercellular communication [49]. Admittedly, we still do not fully understand how the CTLH complex works in higher organisms.

## 3. CTLH Complex Members: Tumor Suppressors or Oncogenes? Drivers or Passengers in Tumorigenesis?

Published data do not definitely respond to the question whether the CTLH proteins are favoring or opposing tumor development and progression. Studied as single proteins, GID4 and RANBP10 have no known role in cancer. For MAEA, MKLN1, RMND5A, and RMND5B, evidence is quite limited and contrasting. Data relative to ARMC8, GID8, and WDR26 indicate a general protumorigenic role of theirs. Finally, RANBP9 is the most studied of the group, but evidence is conflicting.

Altogether, published data support more convincingly a protumorigenic role rather than the contrary for the CTLH complex taken as one functional unit (as discussed below). This role is exerted through a gain of function based on increased expression in tumors compared to the normal tissue of origin. On the other hand, evidence does not support a role as driver of tumorigenesis in the classical sense that refers to genes that when mutated can lead to tumor development. However, widespread increased expression in tumors suggests that they are important for cancer growth and preliminary findings indicate that they could constitute a novel nononcogene addiction [61–63].

### 3.1. Evidence in support of a Tumor Suppressive Role of CTLH Proteins

As mentioned, RANBP9 is the most studied CTLH protein. Correlative studies performed in human samples, but often limited to analysis of its mRNA expression, suggest that reduced expression is associated with worse prognosis. For example, reduced expression of RANBP9 associated with distant metastasis and chemoresistance in gastric cancer [64]. Low levels of RANBP9 transcript also correlate with increased survival in early-stage lung cancer [65].

The study by Qin et al. recently reported that both RANBP9 mRNA and protein expression are increased in colorectal cancer compared to paired normal mucosa. However, *in vitro* and in xenografts from HCT116 and HT29 knockdown of RANBP9 resulted in increased proliferation [66].

The Schild-Poulter's group has reported that RANBP9 inhibits ERK signaling by decreasing the protein levels of c-RAF [67–69]. RANBP9 has also been shown to favor apoptosis [70, 71] and stabilize known tumor suppressors such as p73 and human lethal giant larvae homolog 1 [72, 73]. Thereby, RANBP9 has been proposed to function as a tumor suppressor itself [70, 71, 73]. Finally, RANBP9 bound to TSSC3 (tumor-suppressing STF cDNA3) inhibited anchorage-independent growth and promoted anoikis in osteosarcoma cells [74]. Somewhat in agreement with this role in promoting apoptosis and decreasing survival is the report showing that RANBP9 can decrease the NFκB signaling pathway [75].

MKLN1 was found to represent a novel candidate glioblastoma suppressor gene encompassed within homozygously deleted loci [76]. The interaction between MKLN1 and heme oxigenase-1 favors a less aggressive phenotype and supports an antitumoral role in prostate cancer [77].

In addition to being amplified, the RMND5A gene locus at 2p11.1 has been shown to be also deleted or in a variety of cancers [44]. In the same study, overexpression of RMND5A or RMND5B caused ubiquitination and decrease of the nuclear levels of the known prostatic tumor suppressor NKX3.1 [44]. Also, RMND5A might be a putative tumor suppressor as a strong candidate target of miR-21 in human hepatocellular carcinoma [78]. Finally, the RMND5B locus at 5q35.3 undergoes frequent loss of heterozygosity in breast tumors from BRCA1 and BRCA2 mutation carriers and is located within an uncharacterized prostate cancer heritability locus [44].

### 3.2. Evidence in support of a Protumorigenic Role of CTLH Proteins

In the literature, evidence supporting a protumorigenic effect of the CTLH complex is more conspicuous and convincing than data supporting tumor suppression. Overall, a picture emerges in which overexpression of CTLH genes influences all the main aspects relating to cancer cell plasticity. In summary, (A) CTLH member gene alterations collected from the top 5 most prevalent malignancies indicate that, despite some degree of tissue specificity, mutations are not frequent. On the other hand, copy number gains are present in about 13% of samples. Importantly, increased expression is pervasive. (B) These data are consistent with published articles showing the association of increased expression of single CTLH genes with advanced/aggressive disease. (C) Mechanistically, overexpression of CTLH proteins positively regulates key tumorigenic signaling pathways and (D) regulates cell adhesion and migration. Finally, (E) increased expression correlates with augmented resistance to therapy.

#### 3.2.1. Gene Alterations of the CTLH Complex in Cancer

**Figure 3:**
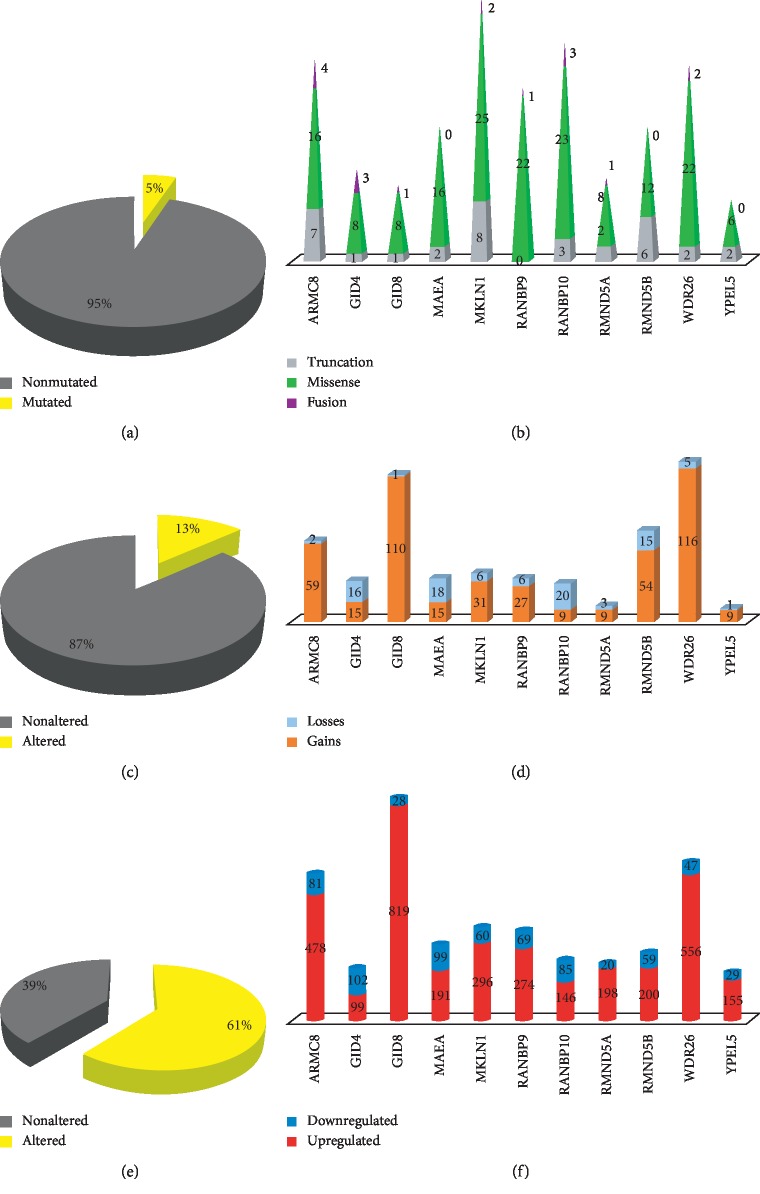
CTLH gene alterations in the most prevalent malignancies in the USA. The TCGA collection of the top 5 most prevalent malignancies in the USA reported in Table 2 was queried for alterations pertaining the 11 CTLH genes (http://www.cbioportal.org). (a) About 5% of cases show mutations of the CTLH genes. (b) For all the analyzed genes, the vast majority of mutations are missense and their functional significance is unknown. (c) 13% of cases show putative copy number variations (CNVs) of the CTLH genes. (d) Putative amplifications are overwhelmingly more prevalent than copy number losses of ARMC8, GID8, RMND5B, WDR26, and YPEL5. On the other hand, GID4, MAEA, and RANBP10 display more copy number losses than gains. (e) 61% of cases show alteration of expression concerning the CTLH genes. (f) Cases of overexpression are overwhelmingly more prevalent than underexpression with the exception of GID4.

A straight tumor suppressive role is in stark contrast with the general observation of an increase in the expression of these proteins in the vast majority of cancers. For this review article, we queried the PanCancer Atlas datasets of the 5 most prevalent cancers in the United States (TCGA: http://www.cbioportal.org) (Table 2) for mutations, copy number variations, and alterations of expression of the 11 CTLH genes. Out of 3,665 surveyed patients, we found a total of 185 mutated cases (5.0%; Figure 3(a)).

However, the vast majority of reported mutations are missense of unknown significance compared to truncations and fusions (Figure 3(b)). Therefore, single base-pair mutations with significant functional consequences might be markedly less than the number recorded. Furthermore, none of the CTLH complex member genes display hotspots or a high number of recurrent mutations (not shown). In spite of reported CTLH single-nucleotide polymorphisms or mutations causing brain developmental disorders and mental retardation, there are no reports of mutations causing or associated with cancer pathogenesis.

Our survey also shows that copy number variations (CNVs) of CTLH genes are only present in about 13.0% of cases (Figure 3(c)). However, gains are consistently more prevalent than losses. Strikingly, GID8 is amplified 110 times while lost only once (Figure 3(d)). Finally, more than 60% of all tumor cases present alterations of CTLH gene expression (Figure 3(e)). Overexpression is markedly predominant. GID8 shows increased expression in 819 instances compared to only 28 cases of reported underexpression (Figure 3(f)). However, a more accurate and systematic analysis with proper statistical consideration will be required to establish how significant and relevant are these alterations in cancer.

#### 3.2.2. Specific CTLH Gene Expression Is Increased in Aggressive Disease

In addition to TCGA data, a number of low-throughput studies reported high expression of specific CTLH genes associated with more aggressive disease and worse prognosis in different types of cancers. These investigations, which only occasionally take into consideration protein levels, suggest that tissue specificity exists in that some CTLH members are more expressed in specific types of tumors.

Although only in a limited number of publications, GID8 has been invariably reported as overexpressed and correlating with advanced disease and poor prognosis. This is consistent with an overexpression in the vast majority of tumors independently of the tissue of origin shown in the TCGA data above (Figure 3).

In fact, GID8 was shown to be significantly upregulated in colorectal cancer and its nuclear levels were inversely correlated with prognosis [79]. GID8-increased expression is also predictive of poor prognosis in gastric cancer [80].

Despite the *in vitro* evidence showing that knockdown of RANBP9 results in increased tumor cell proliferation, invariably, tumors of different types display higher levels of expression compared to the normal counterpart. This is true for lung cancer [65, 81], colorectal cancer [66], osteosarcoma [82], gastric cancer [64], and invasive breast cancer [83].

ARMC8 is probably the second most investigated member of the CTLH complex in cancer after RANBP9. It has been found to be overexpressed in many cancers to the point that it has been proposed as a prognostic marker and/or potential valid target for therapy [84]. In NSCLC, ARMC8 level was significantly higher in tumors than in the adjacent normal tissues and was significantly associated with TNM stage, lymph node metastasis, and poor prognosis. ARMC8 downregulation by siRNA knockdown inhibited growth, colony formation, and invasion, while ARMC8 overexpression had opposite effects [85, 86]. In 206 cases of colon cancer and the matched adjacent normal tissue, ARMC8 has been found to be significantly higher in the membrane and cytoplasm of tumor cells in comparison with the adjacent normal tissues. Furthermore, ARMC8 increased expression associated with aggressive disease and directly related to TNM stage, lymph node metastasis, and poor prognosis. *In vitro*, ARMC8 promoted invasiveness and migration of colon cancer cells and downregulation of its levels had again opposite effects [87].

Knockdown of ARMC8 significantly inhibited osteosarcoma cell proliferation *in vitro*, and it also inhibited xenograft tumor growth *in vivo*. ARMC8 silencing inhibited the migration and invasion of osteosarcoma cells as well [88]. In breast carcinoma where ARMC8 expression was detected mainly in the cytoplasm with occasional membrane immunostaining, infiltrating breast carcinoma showed high expression of ARMC8. Further, higher ARMC8 expression was found to be linked to lymph node metastasis and advanced tumor-node-metastasis stages. Results also indicated that elevated expression of ARMC8 may be involved in atypia-to-carcinoma progression of breast carcinoma [89]. ARMC8 has been reported to promote the malignant progression of ovarian cancer too [90]. The study in question obtained findings similar to those for NSCLC and colon cancer in regard to the association with aggressive disease and *in vitro* oncogenic effects when overexpressed [90]. Similar results are also reported about ARMC8 in hepatocellular carcinoma [91].

That WDR26 has been shown to play a distinct role in breast cancer in a study by Ye et al. is consistent with our survey of TCGA data showing that WDR26 is the CTLH gene with the highest number of amplifications and levels of overexpression in that malignancy [92]. That investigation showed that WDR26 overexpression correlates with shortened survival of breast cancer patients. In addition, downregulation of WDR26 in highly malignant cell lines alleviated GPCR-stimulated PI3-kinase/AKT signaling, tumor cell growth, migration, and invasion but did not alleviate EGF receptor-stimulated PI3-kinase/AKT signaling and tumor cell growth, migration, and invasion. The overexpression of WDR26 had the opposite effect. Collectively, these results identified WDR26 as a potential therapeutic target for breast cancer [92].

RMND5A has also been shown to be a novel potential prognostic marker in breast cancer with higher transcript levels correlating to worse prognosis [93].

Finally, YPEL5 has been shown to be involved in cell division localizing at the spindle during mitosis. Downregulation of YPEL5 leads to diminished cell proliferation [34, 94].

#### 3.2.3. Overexpression of CTLH Proteins Positively Regulates Key Tumorigenic Signaling Pathways

GID8 not only promoted proliferation of colon cancer cells, but its depletion reduced cancer cell growth and expression of WNT-dependent genes [79]. Mechanistically, GID8 was shown to be required for nuclear accumulation of *β*-catenin when WNT canonical signaling is turned on. Considering that the hyperactivation of WNT/*β*-catenin signaling is a major cause of human colorectal cancer and is linked to tumor-initiating cells renewal, these results indicate a major role for GID8 in the pathogenesis of colorectal cancer. In addition, this establishes GID8 as a potential enhancer of WNT signaling in any type of cancer where this pathway is hyperactivated [22, 23].

ARMC8 downregulation in bladder cancer cells inhibited the TGF*β*1-induced migration and invasion and suppressed the EMT progress. Furthermore, ARMC8 silencing inhibited the TGF*β*1-induced expression of *β*-catenin, cyclin D1, and c-myc [95]. Therefore, although via a different signaling pathway, ARMC8 seems to affect some of the same major targets of the WNT signaling.

Interestingly, WDR26 binds Axin1 and negatively regulates *β*-catenin signaling favoring its ubiquitination [96]. On the other hand, WDR26 has been described to be a scaffolding protein involved in various signaling pathways [92, 97, 98]. In breast cancer cells, WDR26 fosters assembly of a specific signaling complex consisting of G*βγ*, PI3-kinase, and AKT2. In an orthotopic MDA-MB231 xenograft model, overexpression of WDR26 mutants in cells caused a disruption in the formation of this complex and abrogated PI3-kinase/AKT activation, tumor cell growth, and metastasis.

This connection between the CTLH complex, WNT signaling, and TGF*β*1 signaling is intriguing. While the essential protein for the formation of the complex GID8 has been shown to promote WNT signaling through nuclear retention of *β*-catenin [79], WDR26 was shown to be a negative regulator of the pathway [96]. On the other hand, ARMC8 has been reported to enhance the TGF*β*1-induced expression of *β*-catenin [95].

RANBP9 has been shown to be able to curb the NFκB signaling-stimulated TGF*β*1 [99]. On the other hand, at the cell membrane, RANBP9 was identified as a factor able to positively regulate the RAS-RAF-MEK kinase pathway when initiated by known kinase oncogenes such as c-MET [67], Axl [100], TRKA [101], and TRKB [102], or c-KIT [57], for example.

Therefore, a scenario emerges in which the CTLH complex in different configurations can modulate positively or negatively WNT signaling. Fascinating is the hypothesis that the scaffold provided by WDR26 can represent a central node integrating signaling of WNT, TGF*β*, and PI3-kinase pathways [92, 97, 98]. The centrality of these highly interconnected pathways in the modulation of stemness and plasticity of cancer cells has been widely demonstrated [2, 4, 10, 15, 25, 103–105]. Indeed, it is tempting to speculate that the of the CTLH complex in its different configurations could act as rheostat of these biological processes.

#### 3.2.4. CTLH Proteins Modulate Cell Adhesion and Migration

Several studies on ARMC8, MKLN1, RANBP9, and RMND5A alone or in combination with each other reported important roles for these CTLH proteins in cell adhesion and migration, which together with EMT-MET are fundamental processes for the establishment of metastatic lesions from primary tumors [106]. Studies on RANBP9 have revealed multiple and important functional links of this scaffold protein with adhesion molecules. Some of these interactions such as the ones with LFA1 [107], obscurin and titin [108], or cofilin [54, 109, 110] have been tested in noncancer contexts. The interaction of RANBP9 with L1-CAM, which is involved in pancreatic cancer pathogenesis, regulates the MAPK signaling activation [111]. RANBP9 influences function and signaling of other integrins, which are major players in virtually all types of malignancies [65, 107, 112]. In this regard, RANBP9 has an important role in integrin-dependent focal adhesion [112].

In cancer cells *in vitro*, generally it has been shown that downmodulation of RANBP9 results in decreased cell adhesion but more invasion ability, while overexpression has opposite effects [64, 65, 113]. A more mechanistic study has shown that RANBP9 can increase migration by inhibiting DYRK1B an inhibitor of migration [114].

As a note of caution for the interpretation of results, RANBP10 has been linked to microtubules dynamics and bears a putative tubulin-binding domain within the *N*-terminus region that is absent in RANBP9 [35, 41, 42, 115]. However, no studies have been performed to test whether Scorpins cross-regulate each other in the context of cell adhesion and migration.

Aside from its participation in the CTLH complex, MKLN1 is known as a multidomain scaffolding intracellular protein that functions in cytoskeletal organization and is strongly implicated in regulation of cell morphology [116–118]. In fact, the duo RANBP9-MKLN1 as such has been reported to regulate cell morphology [117].

Finally, ARMC8 has been isolated together with RANBP9 and MKLN1 in a study where they mediate cell-spreading responses to the thrombospondin-1, a matrix adhesion molecule [116, 119]. Functionally, ARMC8 has been shown to interact with proteins of cell adhesion structures like desmosomes [47, 91]. ARMC8 binds to different catenins including *α*- and *β*-catenin having a significant role in regulating cell migration, proliferation, tissue maintenance, signal transduction, and tumorigenesis [88, 120]. ARMC8 promotes disruption of E-cadherin complex through the regulation of *α*-catenin degradation. This indicated that ARMC8 could regulate cancer invasion through E-cadherin/catenin complex in addition to the fact that it was proposed as potential cancer marker in hepatocellular carcinoma [91].

RMND5A has been shown to be involved in microtubule dynamics, cell migration, nucleokinesis, and chromosome segregation [93].

Overall, these interactions support the hypothesis that the CTLH complex could be an adaptor between the cell adhesion machinery and different cell signaling pathways.

#### 3.2.5. CTLH Complex and Cancer Resistance to Therapy

In a retrospective clinical study, we have recently shown that the levels of RANBP9 protein are inversely correlated with non-small cell lung cancer patient survival in a cohort of patients treated with platinum-based drugs [81]. Important from a therapeutic perspective, we had previously shown that RANBP9 participates in the DNA damage repair [121]. The protein bears several consensus motifs putative target sites of the major kinases involved in the DDR. Active ataxia telangiectasia mutated (ATM), the pinnacle kinase of the DDR, phosphorylates RANBP9 on at least two serine residues and that ATM-kinase activity is required for nuclear accumulation of the protein early after exposure to ionizing radiation [121]. Our results are in agreement with large-scale studies in which both RANBP9 and RANBP10 peptides were found to be phosphorylated following cell exposure to different types of genotoxic stress [122, 123]. In our study, we also unveiled an exquisite sensitivity of NSCLC cells in the absence of RANBP9 to PARP (poly-ADP-ribose polymerase) inhibitors commonly used in the clinics [81]. Knockdown of RANBP9 was also reported to sensitize gastric cancer cells to methotrexate [64].

Interestingly, increased expression of YPEL5 has been reported in erlotinib-treated EGFR-mutant NSCLC [124].

## 4. Concluding Remarks and Future Perspectives

Despite the significant level of conservation throughout evolution, which is indicative of critical biological functions, the CTLH complex is poorly investigated and the knowledge about it is limited especially in the context of cancer [36]. However, CTLH proteins appear to be linked to fundamental biological processes such as proliferation, survival, regulated cell death, cell adhesion, cell migration, and DNA damage response [35, 49, 125]. Furthermore, this complex seems to respond to various types of extracellular cues and regulate major oncogenic pathways [49, 125].

Piecing together all the available tiles of the puzzle, the overall increased expression in human malignancies of the CTLH complex appears to exert essential functions that become even more important for highly proliferative and stressed tumor cells. In light of the links to multiple pathways, a better molecular knowledge of the complex and understanding the reasons why this complex is upregulated in cancer would lay the foundation to target this structure in a precise manner and ablate functions or network connections that enable cancer cell plasticity. To this aim, it will be necessary to gain a better understanding of the molecular functional intricacies of the structure. For example, the duplication during evolution of Gid1 and Gid2 suggests that maybe RANBP10 and RMND5B provide an extra layer of fine-tuning and negative regulation of RANBP9 and RMND5A, respectively. The existence of these paralogs can potentially explain part of the conflicting phenotypes provided by the existing literature about the role of the CTLH protein in cancer. An alternative explanation to conflicting results may relate to some tissue specificity that seems to emerge from the survey of the TCGA collection data. However, another plausible explanation for the apparent conundrum is that this ubiquitously expressed protein complex is instrumental to maintaining cellular homeostasis and therefore is tumor suppressive during early phases of tumorigenesis. At later stages, once a tumor has been established, the same antitumor functions become advantageous for neoplastic growth. Indeed, this distinct opposite effects (tumor suppressors and protumorigenic) are typical of proteins involved in DNA damage response or TGF*β* or autophagy, for example [126–128].

In regards to cell plasticity, we can speculate that proteins and complexes functioning as enablers such as the CTLH one should be not only always present but also highly expressed in their functional form (not mutated) in cancer in order to maintain their intact advantageous functions. On the other hand, their expression can be regulated in a timely fashion similar to what happens to proteins linked to EMT and its reverse process MET. In this regard, an area that requires exploration is the regulation of expression of CTLH gene by microRNA. In fact, RANBP9 was identified in lung cancer as a target of mir-200c, one of the major players [129]. Consistent with the idea that RANBP9 can be dynamically modulated during metastasis is also the prediction that the 3′-UTR is targeted by other established players of EMT such as mir-200a/b/c (http://www.targetscan.org). In the end, this type of posttranscriptional regulation would be also able to explain why RANBP9 mRNA and protein levels not always correlate [81].

For translational purposes, it will be absolutely necessary to understand the links that the CTLH complex have with signaling pathways that are major drivers of oncogenesis. In fact, this complex appears to be potentially linked to all the hallmarks of cancer [130]. Multiple connections with key oncogenic signaling pathways such as PI3-kinase, TGF-*β*, and WNT, for example, suggest an ability to integrate and coordinate these events. These are the same pathways at the heart of stemness and tumor aggressiveness [25, 131].

An important focus of future investigations should also be the identification of the types of stress to which this complex reacts. This is necessary in order to identify potential weaknesses created by targeting the complex or its members. To this aim, we found that depletion of RANBP9 in lung cancer cells renders them more sensitive to genotoxic drugs such as cisplatin and inhibitors of PARP. Considering the centrality of the DDR in cancer, RANBP9 becomes of extreme interest especially in those types of malignancies, characterized by high genomic instability and mutational burden [132]. Therefore, targeting of RANBP9 can be considered as a possible strategy to treat specific types of cancers in which genotoxic drugs are used.

Finally, in order to begin to answer to all the complex questions about the biological functions of the complex and its role in tumorigenesis, new *in vivo* models to conditionally delete or overexpress the CTLH proteins in specific tissues and cell types will be required.

## Figures and Tables

**Table 1:** CTLH proteins, gene chromosomal location, and yeast homologs.

			Uniprot protein ID	Length (aa)	Chromosome cytoband	*S. cerevisiae* homolog
1	ARMC8	Armadillo repeat-containing 8	Q8IUR7	673	3q22.3	Gid5
2	GID4	Glucose-induced degradation protein 4 homolog	Q8IVV7	300	17p11.2	Gid4
3	GID8	Glucose-induced degradation protein 8 homolog	Q9NWU2	228	20q13.33	Gid8
4	MAEA	Macrophage erythroblast attacher	Q7L5Y9	396	4p16.3	Gid9
5	MKLN1	Muskelin 1	Q9UL63	735	7q32.3	Gid7
6	RANBP9	Ran binding protein 9	Q96S59	729	6p23	Gid1
7	RANBP10	Ran binding protein 10	Q6VN20	620	16q22.1	Gid1
8	RMND5A	Required for meiotic nuclear division 5 A	Q9H871	391	2p11.2	Gid2
9	RMND5B	Required for meiotic nuclear division 5 B	Q96G75	393	5q35.3	Gid2
10	WDR26	WD repeat domain-containing protein 26	Q9H7D7	661	1q42.12	Gid7
11	YPEL5	Yippie-like 5	P62699	121	2p23.1	Moh1

The CTLH complex includes 11 known members. We report the protein ID from the Uniprot database, the length in amino acids (aa), the chromosomal location (from the UCSC database; human assembly Dec 2013 = GRCh38/hg38), and the recognized or putative yeast homolog.

**Table 2:** PanCancer studies from the TCGA collection (http://www.cbioportal.org) analyzed for CTLH gene alteration.

			No. of complete tumors analyzed
1	Lung cancer	Lung adenocarcinoma (LUAD)	503
		Lung squamous cell carcinoma (LUSC)	466

2	Prostate cancer	Prostate adenocarcinoma (PRAD)	487

3	Breast cancer	Invasive breast carcinoma (BRCA)	994

4	Colorectal cancer	Colon adenocarcinoma (COADREAD)	524

5	Renal cancer	Kidney chromophobe (KICH)	65
		Kidney renal clear cell carcinoma (KIRC)	352
		Kidney renal papillary cell carcinoma (KIRP)	274

		Total	3665

Studies represent the top 5 most prevalent malignancies in the USA. Number of unique samples is indicated.

## References

[1] Meacham C. E., Morrison S. J. (2013). Tumour heterogeneity and cancer cell plasticity. *Nature*.

[2] Sheng S., Margarida Bernardo M., Dzinic S. H., Chen K., Heath E. I., Sakr W. A. (2018). Tackling tumor heterogeneity and phenotypic plasticity in cancer precision medicine: our experience and a literature review. *Cancer and Metastasis Reviews*.

[3] Vicente-Duenas C., de Diego J., Rodriguez F., Jimenez R., Cobaleda C. (2009). The role of cellular plasticity in cancer development. *Current Medicinal Chemistry*.

[4] da Silva-Diz V., Lorenzo-Sanz L., Bernat-Peguera A., Lopez-Cerda M., Muñoz P. (2018). Cancer cell plasticity: impact on tumor progression and therapy response. *Seminars in Cancer Biology*.

[5] Hölzel M., Bovier A., Tüting T. (2013). Plasticity of tumour and immune cells: a source of heterogeneity and a cause for therapy resistance?. *Nature Reviews Cancer*.

[6] Marjanovic N. D., Weinberg R. A., Chaffer C. L. (2013). Cell plasticity and heterogeneity in cancer. *Clinical Chemistry*.

[7] Tata P. R., Chow R. D., Saladi S. V. (2018). Developmental history provides a roadmap for the emergence of tumor plasticity. *Developmental Cell*.

[8] Diepenbruck M., Christofori G. (2016). Epithelial-mesenchymal transition (EMT) and metastasis: yes, no, maybe?. *Current Opinion in Cell Biology*.

[9] Jolly M. K., Tripathi S. C., Somarelli J. A., Hanash S. M., Levine H. (2017). Epithelial/mesenchymal plasticity: how have quantitative mathematical models helped improve our understanding?. *Molecular Oncology*.

[10] Te Boekhorst V., Friedl P. (2016). Plasticity of cancer cell invasion-mechanisms and implications for therapy. *Molecular and Cellular Basis of Metastasis: Road to Therapy*.

[11] Jolly M. K. (2015). Implications of the hybrid epithelial/mesenchymal phenotype in metastasis. *Frontiers in Oncology*.

[12] Jolly M. K., Mani S. A., Levine H. (2018). Hybrid epithelial/mesenchymal phenotype(s): the “fittest” for metastasis?. *Biochimica et Biophysica Acta (BBA)—Reviews on Cancer*.

[13] Jolly M. K., Somarelli J. A., Sheth M. (2019). Hybrid epithelial/mesenchymal phenotypes promote metastasis and therapy resistance across carcinomas. *Pharmacology & Therapeutics*.

[14] Varga J., De Oliveira T., Greten F. R. (2014). The architect who never sleeps: tumor-induced plasticity. *FEBS Letters*.

[15] Faurobert E., Bouin A.-P., Albiges-Rizo C. (2015). Microenvironment, tumor cell plasticity, and cancer. *Current Opinion in Oncology*.

[16] Davies A. E., Albeck J. G. (2018). Microenvironmental signals and biochemical information processing: cooperative determinants of intratumoral plasticity and heterogeneity. *Frontiers in Cell and Developmental Biology*.

[17] Poli V., Fagnocchi L., Zippo A. (2018). Tumorigenic cell reprogramming and cancer plasticity: interplay between signaling, microenvironment, and epigenetics. *Stem Cells International*.

[18] Friedl P., Alexander S. (2011). Cancer invasion and the microenvironment: plasticity and reciprocity. *Cell*.

[19] Terry S., Zaarour R. F., Venkatesh G. H. (2018). Role of hypoxic stress in regulating tumor immunogenicity, resistance and plasticity. *International Journal of Molecular Sciences*.

[20] Turdo A., Veschi V., Gaggianesi M. (2019). Meeting the challenge of targeting cancer stem cells. *Frontiers in Cell and Developmental Biology*.

[21] Tang D. G. (2012). Understanding cancer stem cell heterogeneity and plasticity. *Cell Research*.

[22] Zhu P., Fan Z. (2018). Cancer stem cells and tumorigenesis. *Biophysics Reports*.

[23] Katoh M. (2017). Canonical and non-canonical WNT signaling in cancer stem cells and their niches: cellular heterogeneity, omics reprogramming, targeted therapy and tumor plasticity (review). *International Journal of Oncology*.

[24] Takebe N., Miele L., Harris P. J. (2015). Targeting Notch, Hedgehog, and Wnt pathways in cancer stem cells: clinical update. *Nature Reviews Clinical Oncology*.

[25] Koury J., Zhong L., Hao J. (2017). Targeting signaling pathways in cancer stem cells for cancer treatment. *Stem Cells International*.

[26] Easwaran H., Tsai H.-C., Baylin S. B. (2014). Cancer epigenetics: tumor heterogeneity, plasticity of stem-like states, and drug resistance. *Molecular Cell*.

[27] Liu H., Pfirrmann T. (2019). The Gid-complex: an emerging player in the ubiquitin ligase league. *Biological Chemistry*.

[28] Maitland M. E. R., Onea G., Chiasson C. A. (2019). The mammalian CTLH complex is an E3 ubiquitin ligase that targets its subunit muskelin for degradation. *Scientific Reports*.

[29] Santt O., Pfirrmann T., Braun B. (2008). The yeast GID complex, a novel ubiquitin ligase (E3) involved in the regulation of carbohydrate metabolism. *Molecular Biology of the Cell*.

[30] Brown C. R., Liu J., Hung G.-C., Carter D., Cui D., Chiang H.-L. (2003). The vid vesicle to vacuole trafficking event requires components of the SNARE membrane fusion machinery. *Journal of Biological Chemistry*.

[31] Lampert F., Stafa D., Goga A. (2018). The multi-subunit GID/CTLH E3 ubiquitin ligase promotes cell proliferation and targets the transcription factor Hbp1 for degradation. *eLife*.

[32] Francis O., Baker G. E., Race P. R., Adams J. C. (2017). Studies of recombinant TWA1 reveal constitutive dimerization. *Bioscience Reports*.

[33] de Araujo T. S., Almeida M. S. (2018). ^1^H, ^13^C and ^15^N chemical shift assignment of lissencephaly-1 homology (LisH) domain homodimer of human two-hybrid-associated protein 1 with RanBPM (Twa1). *Biomolecular NMR Assignments*.

[34] Hosono K., Noda S., Shimizu A. (2010). YPEL5 protein of the YPEL gene family is involved in the cell cycle progression by interacting with two distinct proteins RanBPM and RanBP10. *Genomics*.

[35] Palmieri D., Tessari A., Coppola V. (2018). Scorpins in the DNA damage response. *International Journal of Molecular Sciences*.

[36] Tomastikova E., Cenklová V., Kohoutová L. (2012). Interactions of an Arabidopsis RanBPM homologue with LisH-CTLH domain proteins revealed high conservation of CTLH complexes in eukaryotes. *BMC Plant Biology*.

[37] Francis O., Han F., Adams J. C. (2013). Molecular phylogeny of a RING E3 ubiquitin ligase, conserved in eukaryotic cells and dominated by homologous components, the muskelin/RanBPM/CTLH complex. *PLoS One*.

[38] Wang D., Li Z., Schoen S. R., Messing E. M., Wu G. (2004). A novel MET-interacting protein shares high sequence similarity with RanBPM, but fails to stimulate MET-induced Ras/Erk signaling. *Biochemical and Biophysical Research Communications*.

[39] Bao J., Tang C., Li J. (2014). RAN-binding protein 9 is involved in alternative splicing and is critical for male germ cell development and male fertility. *PLoS Genetics*.

[40] Puverel S., Barrick C., Dolci S., Coppola V., Tessarollo L. (2011). RanBPM is essential for mouse spermatogenesis and oogenesis. *Development*.

[41] Kunert S., Meyer I., Fleischhauer S. (2009). The microtubule modulator RanBP10 plays a critical role in regulation of platelet discoid shape and degranulation. *Blood*.

[42] Meyer I., Kunert S., Schwiebert S. (2012). Altered microtubule equilibrium and impaired thrombus stability in mice lacking RanBP10. *Blood*.

[43] Braun B., Pfirrmann T., Menssen R., Hofmann K., Scheel H., Wolf D. H. (2011). Gid9, a second RING finger protein contributes to the ubiquitin ligase activity of the gid complex required for catabolite degradation. *FEBS Letters*.

[44] Louw A. T., Harvey J., Bentel J. M. (2013). Regulation of NKX3.1 by RMND5 proteins. *Cancer Research*.

[45] Malovannaya A., Lanz R. B., Jung S. Y. (2011). Analysis of the human endogenous coregulator complexome. *Cell*.

[46] Texier Y., Toedt G., Gorza M. (2014). Elution profile analysis of SDS-induced subcomplexes by quantitative mass spectrometry. *Molecular & Cellular Proteomics*.

[47] Gul I. S., Hulpiau P., Sanders E., van Roy F., van Hengel J. (2018). Armc8 is an evolutionarily conserved armadillo protein involved in cell-cell adhesion complexes through multiple molecular interactions. *Bioscience Reports*.

[48] Martinez M. J., Roy S., Archuletta A. B. (2004). Genomic analysis of stationary-phase and exit in *Saccharomyces cerevisiae*: gene expression and identification of novel essential genes. *Molecular Biology of the Cell*.

[49] Salemi L. M., Maitland M. E. R., McTavish C. J., Schild-Poulter C. (2017). Cell signalling pathway regulation by RanBPM: molecular insights and disease implications. *Open Biology*.

[50] Her L.-S., Mao S.-H., Chang C.-Y. (2017). miR-196a enhances neuronal morphology through suppressing RANBP10 to provide neuroprotection in huntington’s disease. *Theranostics*.

[51] Skraban C. M., Wells C. F., Markose P. (2017). WDR26 haploinsufficiency causes a recognizable syndrome of intellectual disability, seizures, abnormal gait, and distinctive facial features. *The American Journal of Human Genetics*.

[52] Nassan M., Li Q., Croarkin P. E. (2017). A genome wide association study suggests the association of muskelin with early onset bipolar disorder: implications for a GABAergic epileptogenic neurogenesis model. *Journal of Affective Disorders*.

[53] Woo J. A., Boggess T., Uhlar C. (2015). RanBP9 at the intersection between cofilin and A*β* pathologies: rescue of neurodegenerative changes by RanBP9 reduction. *Cell Death & Disease*.

[54] Woo J. A., Jung A. R., Lakshmana M. K. (2012). Pivotal role of the RanBP9-cofilin pathway in A*β*-induced apoptosis and neurodegeneration. *Cell Death & Differentiation*.

[55] Bae J. S., Kim J. Y., Park B.-L. (2015). Investigating the potential genetic association between RANBP9 polymorphisms and the risk of schizophrenia. *Molecular Medicine Reports*.

[56] Flaumenhaft R. (2009). Getting in shape with RanBP10. *Blood*.

[57] Puverel S., Kiris E., Singh S. (2016). RanBPM (RanBP9) regulates mouse c-Kit receptor level and is essential for normal development of bone marrow progenitor cells. *Oncotarget*.

[58] Javan G. T., Salhotra A., Finley S. J., Soni S. (2018). Erythroblast macrophage protein (Emp): past, present, and future. *European Journal of Haematology*.

[59] Leal-Esteban L. C., Rothé B., Fortier S., Isenschmid M., Constam D. B. (2018). Role of bicaudal C1 in renal gluconeogenesis and its novel interaction with the CTLH complex. *PLoS Genetics*.

[60] Shimizu K., Okamoto M., Terada T. (2017). Adenovirus vector-mediated macrophage erythroblast attacher (MAEA) overexpression in primary mouse hepatocytes attenuates hepatic gluconeogenesis. *Biochemistry and Biophysics Reports*.

[61] Solimini N. L., Luo J., Elledge S. J. (2007). Non-oncogene addiction and the stress phenotype of cancer cells. *Cell*.

[62] Luo J., Solimini N. L., Elledge S. J. (2009). Principles of cancer therapy: oncogene and non-oncogene addiction. *Cell*.

[63] Nagel R., Semenova E. A., Berns A. (2016). Drugging the addict: non-oncogene addiction as a target for cancer therapy. *EMBO Reports*.

[64] Shao S., Sun P. H., Satherley L. K. (2016). Reduced RanBPM expression is associated with distant metastasis in gastric cancer and chemoresistance. *Anticancer Research*.

[65] Zhao Z. (2017). Reduced expression of RanBPM is associated with poorer survival from lung cancer and increased proliferation and invasion of lung cancer cells in vitro. *Anticancer Research*.

[66] Qin C., Zhang Q., Wu G. (2019). RANBP9 suppresses tumor proliferation in colorectal cancer. *Oncology Letters*.

[67] Wang D., Li Z., Messing E. M., Wu G. (2002). Activation of Ras/Erk pathway by a novel MET-interacting protein RanBPM. *Journal of Biological Chemistry*.

[68] Atabakhsh E., Schild-Poulter C. (2012). RanBPM is an inhibitor of ERK signaling. *PLoS One*.

[69] McTavish C. J., Bérubé-Janzen W., Wang X. (2019). Regulation of c-raf stability through the CTLH complex. *International Journal of Molecular Sciences*.

[70] Atabakhsh E., Bryce D. M., Lefebvre K. J., Schild-Poulter C. (2009). RanBPM has proapoptotic activities that regulate cell death pathways in response to DNA damage. *Molecular Cancer Research*.

[71] Liu T., Roh S. E., Woo J. A., Ryu H., Kang D. E. (2013). Cooperative role of RanBP9 and P73 in mitochondria-mediated apoptosis. *Cell Death & Disease*.

[72] Suresh B., Ramakrishna S., Kim Y.-S., Kim S.-M., Kim M.-S., Baek K.-H. (2010). Stability and function of mammalian lethal giant larvae-1 oncoprotein are regulated by the scaffolding protein RanBPM. *Journal of Biological Chemistry*.

[73] Kramer S., Ozaki T., Miyazaki K., Kato C., Hanamoto T., Nakagawara A. (2005). Protein stability and function of p73 are modulated by a physical interaction with RanBPM in mammalian cultured cells. *Oncogene*.

[74] Dai H., Lv Y.-F., Yan G.-N., Meng G., Zhang X., Guo Q.-N. (2016). RanBP9/TSSC3 complex cooperates to suppress anoikis resistance and metastasis via inhibiting Src-mediated Akt signaling in osteosarcoma. *Cell Death & Disease*.

[75] Wang L., Fu C., Cui Y. (2012). The Ran-binding protein RanBPM can depress the NF-kappaB pathway by interacting with TRAF6. *Molecular and Cellular Biochemistry*.

[76] Nord H., Hartmann C., Andersson R. (2009). Characterization of novel and complex genomic aberrations in glioblastoma using a 32K BAC array. *Neuro-Oncology*.

[77] Gueron G., Giudice J., Valacco P. (2014). Heme-oxygenase-1 implications in cell morphology and the adhesive behavior of prostate cancer cells. *Oncotarget*.

[78] Koenig A. B., Barajas J. M., Guerrero M. J., Ghoshal K. (2018). A comprehensive analysis of argonaute-CLIP data identifies novel, conserved and species-specific targets of miR-21 in human liver and hepatocellular carcinoma. *International Journal of Molecular Sciences*.

[79] Lu Y., Xie S., Zhang W. (2017). Twa1/Gid8 is a *β*-catenin nuclear retention factor in Wnt signaling and colorectal tumorigenesis. *Cell Research*.

[80] Xiong J., Feng Z., Li Z. (2019). Overexpression of TWA1 predicts poor prognosis in patients with gastric cancer. *Pathology—Research and Practice*.

[81] Tessari A., Parbhoo K., Pawlikowski M. (2018). RANBP9 affects cancer cells response to genotoxic stress and its overexpression is associated with worse response to platinum in NSCLC patients. *Oncogene*.

[82] Zhu L. L. (2016). Expression of cartilage antitumor component RanBP9 in osteosarcoma. *Journal of Biological Regulators and Homeostatic Agents*.

[83] Emberley E. D., Gietz R. D., Campbell J. D., HayGlass K. T., Murphy L. C., Watson P. H. (2002). RanBPM interacts with psoriasin in vitro and their expression correlates with specific clinical features in vivo in breast cancer. *BMC Cancer*.

[84] Zhou D., Zhang W., Wang Y., Chen L., Luan J. (2016). ARMc8: a potential diagnostic and therapeutic target for cancers. *Human Pathology*.

[85] Amin A., Bukhari S., Mokhdomi T. A. (2015). Comparative proteomics and global genome-wide expression data implicate role of ARMC8 in lung cancer. *Asian Pacific Journal of Cancer Prevention*.

[86] Xie C., Jiang G., Fan C. (2014). ARMC8*α* promotes proliferation and invasion of non-small cell lung cancer cells by activating the canonical Wnt signaling pathway. *Tumor Biology*.

[87] Jiang G., Zhang Y., Zhang X. (2015). ARMc8 indicates aggressive colon cancers and promotes invasiveness and migration of colon cancer cells. *Tumor Biology*.

[88] Jiang F., Shi Y., Lu H., Li G. (2016). Armadillo repeat-containing protein 8 (ARMC8) silencing inhibits proliferation and invasion in osteosarcoma cells. *Oncology Research Featuring Preclinical and Clinical Cancer Therapeutics*.

[89] Fan C., Zhao Y., Mao X. (2014). Armc8 expression was elevated during atypia-to-carcinoma progression and associated with cancer development of breast carcinoma. *Tumor Biology*.

[90] Jiang G., Yang D., Wang L. (2015). A novel biomarker ARMc8 promotes the malignant progression of ovarian cancer. *Human Pathology*.

[91] Zhao Y., Peng S., Jia C., Xu F., Xu Y., Dai C. (2016). Armc8 regulates the invasive ability of hepatocellular carcinoma through E-cadherin/catenin complex. *Tumor Biology*.

[92] Ye Y., Tang X., Sun Z., Chen S. (2016). Upregulated WDR26 serves as a scaffold to coordinate PI3K/AKT pathway-driven breast cancer cell growth, migration, and invasion. *Oncotarget*.

[93] Liu H., Ye H. (2017). Screening of the prognostic targets for breast cancer based co-expression modules analysis. *Molecular Medicine Reports*.

[94] Hosono K., Sasaki T., Minoshima S., Shimizu N. (2004). Identification and characterization of a novel gene family YPEL in a wide spectrum of eukaryotic species. *Gene*.

[95] Liang X., Men Q.-L., Li Y.-w., Li H.-C., Chong T., Li Z.-l. (2017). Silencing of armadillo repeat-containing protein 8 (ARMc8) inhibits TGF-*β*-induced EMT in bladder carcinoma UMUC3 cells. *Oncology Research Featuring Preclinical and Clinical Cancer Therapeutics*.

[96] Goto T., Matsuzawa J., Iemura S.-I., Natsume T., Shibuya H. (2016). WDR26 is a new partner of Axin1 in the canonical Wnt signaling pathway. *FEBS Letters*.

[97] Sun Z., Smrcka A. V., Chen S. (2013). WDR26 functions as a scaffolding protein to promote g*βγ*-mediated phospholipase C *β*2 (PLC*β*2) activation in leukocytes. *Journal of Biological Chemistry*.

[98] Zhu Y., Wang Y., Xia C. (2004). WDR26: a novel G*β*-like protein, suppresses MAPK signaling pathway. *Journal of Cellular Biochemistry*.

[99] Zhang J., Ma W., Tian S. (2014). RanBPM interacts with T*β*RI, TRAF6 and curbs TGF induced nuclear accumulation of T*β*RI. *Cellular Signalling*.

[100] Hafizi S., Gustafsson A., Stenhoff J., Dahlbäck B. (2005). The Ran binding protein RanBPM interacts with Axl and Sky receptor tyrosine kinases. *The International Journal of Biochemistry & Cell Biology*.

[101] Yuan Y., Fu C., Chen H., Wang X., Deng W., Huang B.-R. (2006). The Ran binding protein RanBPM interacts with TrkA receptor. *Neuroscience Letters*.

[102] Yin Y. X., Sun Z. P, Huang S. H, Zhao L, Geng Z, Chen Z. Y (2010). RanBPM contributes to TrkB signaling and regulates brain-derived neurotrophic factor-induced neuronal morphogenesis and survival. *Journal of Neurochemistry*.

[103] Matsui W. H. (2016). Cancer stem cell signaling pathways. *Medicine*.

[104] Karamboulas C., Ailles L. (2013). Developmental signaling pathways in cancer stem cells of solid tumors. *Biochimica et Biophysica Acta (BBA)—General Subjects*.

[105] Beyer T. A., Narimatsu M., Weiss A., David L., Wrana J. L. (2013). The TGF*β* superfamily in stem cell biology and early mammalian embryonic development. *Biochimica et Biophysica Acta (BBA)—General Subjects*.

[106] Lambert A. W., Pattabiraman D. R., Weinberg R. A. (2017). Emerging biological principles of metastasis. *Cell*.

[107] Denti S., Sirri A., Cheli A. (2004). RanBPM is a phosphoprotein that associates with the plasma membrane and interacts with the integrin LFA-1. *Journal of Biological Chemistry*.

[108] Bowman A. L., Catino D. H., Strong J. C., Randall W. R., Kontrogianni-Konstantopoulos A., Bloch R. J. (2008). The rho-guanine nucleotide exchange factor domain of obscurin regulates assembly of titin at the Z-disk through interactions with Ran binding protein 9. *Molecular Biology of the Cell*.

[109] Palavicini J. P., Wang H., Minond D., Bianchi E., Xu S., Lakshmana M. K. (2014). RanBP9 overexpression down-regulates phospho-cofilin, causes early synaptic deficits and impaired learning, and accelerates accumulation of amyloid plaques in the mouse brain. *Journal of Alzheimer’s Disease*.

[110] Roh S.-E., Woo J. A., Lakshmana M. K. (2013). Mitochondrial dysfunction and calcium deregulation by the RanBP9-cofilin pathway. *The FASEB Journal*.

[111] Cheng L., Lemmon S., Lemmon V. (2005). RanBPM is an L1-interacting protein that regulates L1-mediated mitogen-activated protein kinase activation. *Journal of Neurochemistry*.

[112] Woo J. A., Roh S.-E., Lakshmana M. K., Kang D. E. (2012). Pivotal role of RanBP9 in integrin-dependent focal adhesion signaling and assembly. *The FASEB Journal*.

[113] Wei J.-D., Kim J.-Y., Kim A.-K., Jang S. K., Kim J.-H. (2013). RanBPM protein acts as a negative regulator of BLT2 receptor to attenuate BLT2-mediated cell motility. *Journal of Biological Chemistry*.

[114] Zou Y., Lim S., Lee K., Deng X., Friedman E. (2003). Serine/threonine kinase Mirk/Dyrk1B is an inhibitor of epithelial cell migration and is negatively regulated by the Met adaptor Ran-binding protein M. *Journal of Biological Chemistry*.

[115] Schulze H., Dose M., Korpal M., Meyer I., Italiano J. E., Shivdasani R. A. (2008). RanBP10 is a cytoplasmic guanine nucleotide exchange factor that modulates noncentrosomal microtubules. *Journal of Biological Chemistry*.

[116] Adams J. C., Seed B., Lawler J. (1998). Muskelin, a novel intracellular mediator of cell adhesive and cytoskeletal responses to thrombospondin-1. *The EMBO Journal*.

[117] Valiyaveettil M., Bentley A. A., Gursahaney P. (2008). Novel role of the muskelin-RanBP9 complex as a nucleocytoplasmic mediator of cell morphology regulation. *The Journal of Cell Biology*.

[118] Tripathi B. K., Lowy D. R., Zelenka P. S. (2015). The Cdk5 activator P39 specifically links muskelin to myosin II and regulates stress fiber formation and actin organization in lens. *Experimental Cell Research*.

[119] Kobayashi N., Yang J., Ueda A. (2007). RanBPM, Muskelin, p48EMLP, p44CTLH, and the armadillo-repeat proteins ARMC8*α* and ARMC8*β* are components of the CTLH complex. *Gene*.

[120] Suzuki T., Ueda A., Kobayashi N. (2008). Proteasome-dependent degradation of *α*-catenin is regulated by interaction with ARMc8*α*. *Biochemical Journal*.

[121] Palmieri D., Scarpa M., Tessari A. (2016). Ran binding protein 9 (RanBP9) is a novel mediator of cellular DNA damage response in lung cancer cells. *Oncotarget*.

[122] Beli P., Lukashchuk N., Wagner S. A. (2012). Proteomic investigations reveal a role for RNA processing factor THRAP3 in the DNA damage response. *Molecular Cell*.

[123] Matsuoka S., Ballif B. A., Smogorzewska A. (2007). ATM and ATR substrate analysis reveals extensive protein networks responsive to DNA damage. *Science*.

[124] Wu X. (2018). Up-regulation of YPEL1 and YPEL5 and down-regulation of ITGA2 in erlotinib-treated EGFR-mutant non-small cell lung cancer: a bioinformatic analysis. *Gene*.

[125] Das S., Suresh B., Kim H., Ramakrishna S. (2017). RanBPM: a potential therapeutic target for modulating diverse physiological disorders. *Drug Discovery Today*.

[126] Broustas C. G., Lieberman H. B. (2014). DNA damage response genes and the development of cancer metastasis. *Radiation Research*.

[127] Bachman K. E., Park B. H. (2005). Duel nature of TGF-*β* signaling: tumor suppressor vs. tumor promoter. *Current Opinion in Oncology*.

[128] Puissant A., Fenouille N., Auberger P. (2012). When autophagy meets cancer through p62/SQSTM1. *American Journal of Cancer Research*.

[129] Zhang T., Guo L., Creighton C. J. (2016). A genetic cell context-dependent role for ZEB1 in lung cancer. *Nature Communications*.

[130] Hanahan D., Weinberg R. A. (2011). Hallmarks of cancer: the next generation. *Cell*.

[131] Aponte P. M., Caicedo A. (2017). Stemness in cancer: stem cells, cancer stem cells, and their microenvironment. *Stem Cells International*.

[132] Lawrence M. S., Stojanov P., Polak P. (2013). Mutational heterogeneity in cancer and the search for new cancer-associated genes. *Nature*.

